# Clinical utility of tumor-infiltrating lymphocyte evaluation by two different methods in breast cancer patients treated with neoadjuvant chemotherapy

**DOI:** 10.1007/s12282-025-01665-y

**Published:** 2025-01-14

**Authors:** Masayuki Nagahashi, Eri Ishikawa, Takahiro Nagai, Haruka Kanaoka, Aoi Oshiro, Yusa Togashi, Akira Hattori, Junko Tsuchida, Tomoko Higuchi, Arisa Nishimukai, Keiko Murase, Yuichi Takatsuka, Takako Kihara, Yiwei Ling, Shujiro Okuda, Seiichi Hirota, Yasuo Miyoshi

**Affiliations:** 1 Division of Breast and Endocrine Surgery, Department of Surgery, School of Medicine, Hyogo Medical University, 1-1 Mukogawa-cho, Nishinomiya, Hyogo 663-8501 Japan; 2 Department of Diagnostic Pathology, School of Medicine, Hyogo Medical University, Nishinomiya City, Hyogo Japan; 3 Center for Genomic Data Management, Niigata University Medical and Dental Hospital, 1-754 Asahimachi-dori, Chuo-ku, Niigata, 951-8520 Japan; 4 Division of Bioinformatics, Niigata University Graduate School of Medical and Dental Sciences, 2-5274 Gakkocho-dori, Chuo-ku, Niigata, 951-8514 Japan; 5 Medical AI Center, Niigata University School of Medicine, 2-5274 Gakkocho-dori, Chuo-ku, Niigata, 951-8514 Japan

**Keywords:** Breast neoplasm, Tumor-infiltrating lymphocyte, Neoadjuvant chemotherapy, Pathological complete response, Machine learning

## Abstract

**Purpose:**

The aim of this study was to examine the clinical utility of tumor-infiltrating lymphocytes (TILs) evaluated by “average” and “hot-spot” methods in breast cancer patients.

**Methods:**

We examined 367 breast cancer patients without neoadjuvant chemotherapy (NAC) by average and hot-spot methods to determine the consistency of TIL scores between biopsy and surgical specimens. TIL scores before NAC were also compared with the pathological complete response (pCR) rate and clinical outcomes in 144 breast cancer patients that received NAC. TIL scores evaluated by the two methods were predicted from clinicopathological data using random forest regression.

**Results:**

Surgical specimens showed higher TIL scores than biopsy specimens using the hot-spot method (*p* < 0.001), while biopsy and surgical specimens showed similar TIL scores using the average method. There was a linear relationship between the pCR rate and TIL scores determined using hot-spot (*p* < 0.001) and average methods *(p* = 0.001). Patients without pCR and low TILs by the average method had significantly worse overall survival compared to other patients (*p* = 0.02). The root mean squared errors of the predicted TIL score for the test set were 19.662 (hot-spot) and 10.955 (average).

**Conclusion:**

The average method may have an advantage for breast cancer patients receiving NAC, since the TIL score using this method is more consistent between biopsy and surgical specimens, and it associates better with clinical outcomes. Our exploratory study showed that machine learning from clinicopathological data may better predict TIL scores assessed by the average, rather than hot-spot, method.

**Supplementary Information:**

The online version contains supplementary material available at 10.1007/s12282-025-01665-y.

## Introduction

The tumor immune microenvironment has been shown to have a significant influence on the disease process of breast cancer [1]. Tumor-infiltrating lymphocytes (TILs) are immune cells that migrate from the blood to the tumor microenvironment, where they exert a variety of functions [2]. The immune cells found in the tumor and its stroma include not only lymphocytes, such as T cells and B cells, but also natural killer cells, macrophages, neutrophils, and dendritic cells [2, 3]. Although the composition of immune cells varies depending on tumor character, and the function of these immune cells changes dynamically during the tumor development process, TILs often act in a tumor suppressive manner through the function of tumor suppressor cells, especially CD8^+^ T cells [2, 4]. Indeed, it has been reported that increased TILs are associated with a better prognosis in breast cancer patients. Moreover, TILs are associated with the efficacy of chemotherapy, and increased TILs are associated with higher pathological complete response (pCR) rates [5–7]. Furthermore, immune checkpoint inhibitors have recently been introduced in triple-negative breast cancer (TNBC), and the relationship between TILs and the efficacy of immune checkpoint inhibitors has been reported [8, 9]. Thus, in breast cancer, TILs are attracting attention, not only as a prognostic marker, but also as a predictive marker for various therapies.

To assess TILs in breast cancer clinically, the International TILs Working Group introduced a standard method of histopathological evaluation of TILs, which has been widely accepted [10, 11]. In this method, the overall trend of TIL distribution is observed by microscopy, and histopathological analysis is performed to assess the average value of TILs in the tumor stromal area. In addition to this standard method, we have previously focused on hot-spot areas of TILs and proposed a simpler method to evaluate TILs using hot-spots, and have reported its clinical usefulness [7, 12–17]. The advantage of the “hot-spot” method is that it is easy to evaluate differences between cases, even when TILs are very low, as in hormone receptor-positive breast cancer [7, 12–17]. Based on the above, we believe that the “average” method standardized by the International TILs Working Group is the international standard, and that the hot-spot method can also be used to effectively evaluate TILs for clinical use, depending on the particular case.

In recent years, human epidermal growth factor receptor 2 (HER2)-positive breast cancer and TNBC have been increasingly indicated for preoperative chemotherapy with the introduction of the residual disease-guided approach and immune checkpoint inhibitors [18, 19]. Even considering that TILs correlate well with pCR in HER2-positive and TNBCs, the evaluation of TILs utilizing biopsy specimens prior to treatment is expected to be clinically useful. Since a biopsy specimen has a much smaller area than a surgical specimen, this may affect the evaluation of TILs. Although there are many reports evaluating TILs in pre-treatment biopsy specimens and post-treatment surgical specimens, and assessing the clinical utility of each, it has not been adequately verified whether the evaluation of TIL in biopsy specimens is equivalent to that in surgical specimens. The aim of this study was to examine the clinical utility of TILs evaluated by the two different methods of average and hot-spot using biopsy specimens in breast cancer patients who had undergone preoperative chemotherapy. To examine the consistency of TIL assessment in biopsy and surgical specimens, we also studied surgical cases that had not received preoperative treatment. Furthermore, for future development, we aimed to conduct an exploratory study to determine whether it is possible to predict TIL scores using only clinical information, such as blood data and pathological findings obtained in routine clinical practice, without directly measuring TILs.

## Patients and methods

### Patient eligibility

From August 2016 to October 2023, 907 breast cancer patients underwent surgery at Hyogo Medical University Hospital, and the pathological diagnosis was evaluated for each patient. Of these, 286 patients received preoperative therapies, including neoadjuvant chemotherapy (NAC) in 182 patients and neoadjuvant hormone therapy in 104 patients, and the remaining 621 patients underwent surgery without any preoperative therapies. Out of the 621 surgical cases without preoperative therapy, 367 had formalin-fixed paraffin-embedded tissue samples of both biopsy and surgical specimens in storage and available for examination. From the 182 patients receiving NAC, patients with bilateral breast cancer, recurrent cancer, and Stage IV disease with distant metastasis were excluded, leaving 144 patients for whom clinical information was available included in the study. Similarly, patients with bilateral breast cancer, recurrent cancer, and Stage IV disease with distant metastasis were excluded from the 621 surgical cases without preoperative therapy, and 477 patients for whom clinical information was available were included in the prognostic analysis. The Institutional Review Board of the Hyogo Medical University approved this study (No. 1886), which was planned in accordance with the Declaration of Helsinki. Since this study collected only retrospective clinical data and offered no risk to the patients, they were not asked for their written informed consent.

### Pathological diagnosis, immunohistochemistry and subtype classification

All patients included in the study were histologically diagnosed with breast cancer. Immunohistochemistry for estrogen receptor (ER), HER2 and Ki-67 was performed on formalin-fixed paraffin-embedded tumor tissues obtained from biopsy or surgical specimens. Cases with nuclear staining of 1% or more were classified as ER-positive. Cases were also classified as HER2-negative with either an immunohistochemistry score of 0 to 2+ or with no *HER2* amplification, as confirmed by in situ hybridization. Based on immunohistochemistry, we classified ER-positive and HER2-negative breast cancers into luminal A and luminal B according to Ki-67 expression levels (luminal A: Ki-67 < 25%; luminal B: Ki-67 ≥ 25%).

### Pathological evaluation of TILs

Formalin-fixed paraffin-embedded biopsy and surgical specimens were used for preparing hematoxylin and eosin-stained sections, and TIL scores were evaluated under microscopy by the hot-spot method and average method as described previously [7, 10, 11, 13]. Briefly, for the hot-spot method, we microscopically identified lesions containing a relatively high number of invasive cancer cells and lymphocyte infiltration using a low-power field (40×). The hot-spot with the highest lymphocyte infiltration was selected in a medium-power field (100×). Excluding neutrophils, eosinophils, and macrophages, lymphocytes and plasma cells in both the peritumoral and intertumoral stromal regions were evaluated. Finally, the TIL score was calculated as the percentage of area involved in lymphocytes and plasma cells out of the entire tumor and adjacent stroma. For the “average” method, evaluation was performed based on the standardized assessment of TILs in breast cancer by the International Immuno-Oncology Biomarker Working Group (www.tilsinbreastcancer.org) [10, 11]. We estimated the proportion of the area infiltrated by lymphocytes to the area of the entire tumor plus adjacent stroma, and classified the TIL scores as low (< 10%), intermediate (10–50%), or high (≥ 50%) in both methods. TIL scores were independently examined by two investigators, and in cases of discrepancies, they were discussed until a consensus was reached.

### Collection of clinical data for machine learning

For the 621 surgical cases without preoperative therapy, data including preoperative blood parameters, height, weight, date of surgery, date of birth, TNM classification, surgical findings, and pathology findings of resection specimens were collected from the medical records of eligible patients. TIL scores (hot-spot and average) were also obtained.

### Data preprocessing

The data was preprocessed as described below to make it ready for use in machine learning. For blood hemoglobin A1c, National Glycohemoglobin Standardization Program values were used, except in patients where only the Japan Diabetes Society standard values were available, in which case the Japan Diabetes Society values plus 0.4 were used. For numerical data, values that were found to be below the detection limit were treated as the detection limit value itself. For the degree of lymphatic and vascular invasion in pathological findings, the pathologist's grading was converted into numerical values. In cases where the degree of lymphatic or vascular invasion was reported to be absent, the item was set to 0. HER2 immunohistochemistry staining was also converted from a grading system to a numerical value. Age at surgery was calculated from the date of surgery and date of birth. T and N from the TNM classification were converted into numerical values according to the conversion described in Supplementary Table 1. Since tissue histological type is a nominal measure, it was converted to a one-hot vector [20]. Finally, we excluded cases in which the histological type was ≤ 1% of all the 621 cases. As a result, 607 cases remained.

### Learning

Python 3.8 [21] and Scikit-learn 0.24.2 [22] were used for machine learning with random forests. Random forests, developed by Tin Kam Ho [23], Leo Breiman [24], and others, are a new approach to machine learning [23, 24]and involve an ensemble learning algorithm using decision trees.

In the present study, the explanatory variables taken from preoperative blood data were lactate dehydrogenase, carcinoembryonic antigen, carbohydrate antigen 15-3, albumin, low-density lipoprotein cholesterol, triglycerides, hemoglobin A1c, C-reactive protein, white blood cell count, red blood cell count, hemoglobin level, platelet count, ratio of segmented leukocytes, and ratio of lymphocytes. In addition, the following explanatory variables were taken from the pathological findings: degree of lymphatic invasion, vascular invasion, nuclear grade, histological grade, ER status, progesterone receptor status, Ki-67, and HER2-positive status. Other explanatory variables used were height, weight, age at the date of surgery, pT and pN (from the TNM classification) converted to tabulated values, and one-hot vectorized histological type.

From the 607 cases, cases with missing explanatory variables were excluded. As a result, 468 cases remained. The 468 cases were divided into study and test cases at a ratio of 7:3. There were 327 training cases and 141 test cases. A random forest regressor was trained using the 327 training cases as supervised data, with TILs (hot spots) or TILs (average) as the objective variable. RandomForestRegressor from Scikit-learn was used as the random forest regressor.

The regressors after training were used to predict the objective variable for the 141 test cases, and the obtained predictions were compared with the true values to calculate the root mean squared error. The root mean squared error represents the average absolute value of how far the predicted value deviates from the true value. For example, a root mean squared error of 10 implies that if the true value is 40%, the predicted value is expected to be in the range of 30% to 50%.

### Statistical analysis

A chi-squared test was used to compare data on TILs and clinical findings between the two groups or between multiple groups. Kaplan–Meier plots and log-rank tests of disease-free survival (DFS) or overall survival (OS) were applied for each group. Statistical calculations were performed using JMP Pro 17 (SAS Institute Inc., Cary, NC, USA) and a at *p* value < 0.05 was considered to indicated a significant difference with a two-tailed test.

## Results

### Consistency of TIL scores between biopsy and surgical specimens using the hot-spot and average methods

**Fig. 1:**
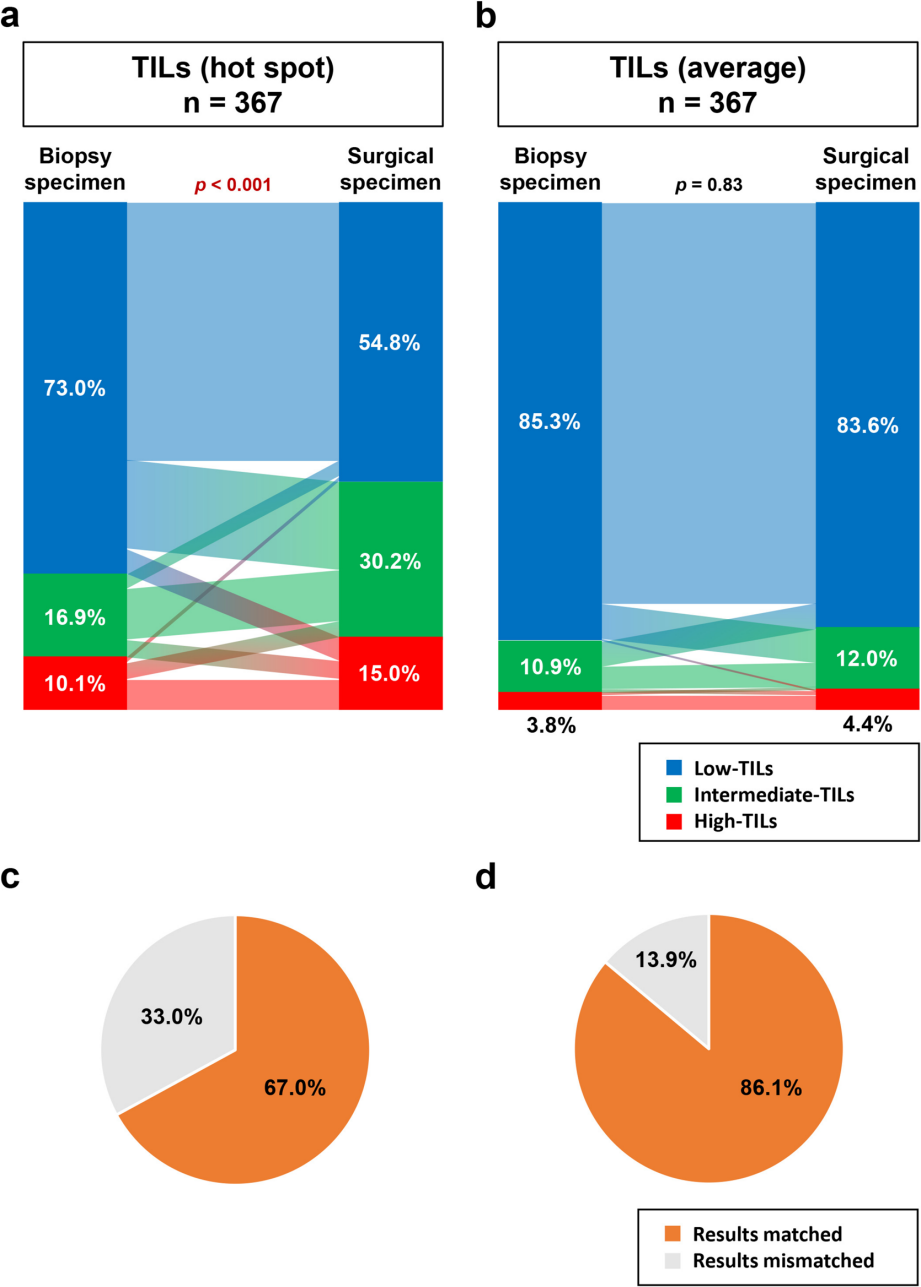
Consistency of tumor-infiltrating lymphocyte (TIL) scores between biopsy and surgical specimens using the “hot-spot” and “average” methods. The percentages of patients with low (blue), intermediate (green) or high (red) TIL scores for biopsy and surgical specimens using the hot-spot (**a**) and average (**b**) methods are shown. Pie chart showing the percentages of patients in whom the TIL scores of biopsy and surgical specimens matched (orange) or mismatched (grey) using the hot-spot (**c**) and average (**d**) methods

To determine the consistency of TIL scores evaluated by the hot-spot or average methods between biopsy and surgical specimens in the same patient, we studied specimens from 367 patients undergoing surgery without preoperative therapy in which both biopsy and surgical specimens were available (Fig. 1a). Using the hot-spot method, surgical specimens showed higher TIL scores than biopsy specimens, resulting in significant differences in the percentages of TIL-score categories (*p* < 0.001; Fig. 1a). On the other hand, biopsy and surgical specimens showed similar percentages of TIL-score categories using the average method (*p* = 0.83; Fig. 1b). The percentage of patients with concordant TIL-score categories for biopsy and surgical specimens (high = high, intermediate = intermediate, or low = low) was 67.0% for the hot-spot method (Fig. 1c) and 86.1% for the average method (Fig. 1d).

### Association between pre-treatment TIL scores and pCR rate in breast cancer patients receiving NAC

**Fig. 2:**
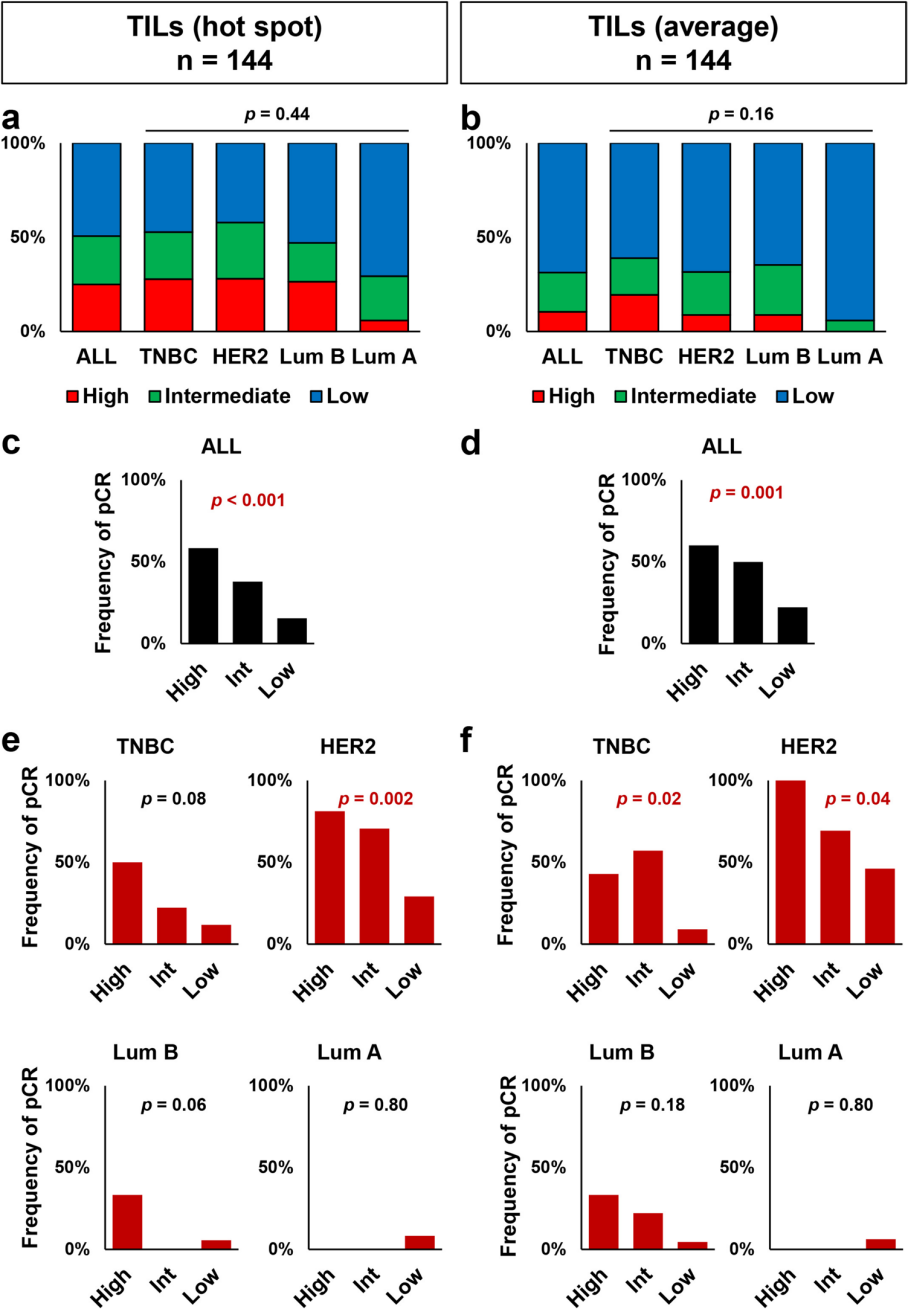
Pre-treatment tumor-infiltrating lymphocyte (TIL) scores (high, intermediate, low) per subtype using the “hot-spot” and “average” methods and the relationship between TIL scores and pathological complete response (pCR) for 144 patients receiving neoadjuvant chemotherapy. The percentages of patients with low (blue), intermediate (green) or high (red) TIL scores for each subtype by hot-spot (**a**) and average (**b**) methods are shown. pCR rates according to TIL scores are shown by hot-spot (**c**) and average (**d**) methods for all patients. pCR rates according to cancer subtype are shown by hot-spot (**e**) and average (**f**) methods. *Int* intermediate, *TNBC* triple-negative breast cancer, *HER2* human epidermal growth factor receptor 2, *Lum* luminal

Next, we evaluated the clinical significance of TIL scores determined by the hot-spot and average methods utilizing biopsy specimens taken for the initial diagnosis in 144 breast cancer patients who underwent NAC. In this patient group, the percentage of patients with high-TILs using the hot-spot method (25.0%) was significantly higher than for the average method (10.4%; *p* < 0.001; Supplementary Fig. 1). The cancer subtypes for the 144 patients receiving NAC were TNBC in 36 patients (25.0%), HER2-positive in 57 (39.6%), luminal B type in 34 (23.6%), and luminal A type in 17 (11.8%). Looking at the data by the TIL evaluation method and subtype, the percentage of patients with high-TILs tended to be lower in the luminal A subtype compared to other subtypes, although there was no significant difference using either the hot-spot method (*p* = 0.44; Fig. 2a) or the average method (*p* = 0.16, Fig. 2b). A linear relationship existed between the pCR rate and TIL scores determined by the hot-spot method (*p* < 0.001, Fig. 2c), as well as the average method (*p* = 0.001, Fig. 2d). According to subtype, the pCR rate was linearly related to hot-spot TIL scores in HER2-positive subtypes (*p* = 0.002) and tended to be related to hot-spot TIL scores in the TNBC and luminal B subtypes (*p* = 0.08 and *p* = 0.06, respectively, Fig. 2e). Moreover, there was a linear relationship between the pCR rates in TNBC and HER2-positive subtypes and TIL scores determined by the average method (*p* = 0.02 and *p* = 0.04, respectively, Fig. 2f). In the luminal A subtype, only a few patients showed pCR, and there was no relationship between TILs and pCR using either the hot-spot or average methodologies (Fig. 2e and f).

### Association between pre-treatment TIL scores and clinical outcomes in breast cancer patients receiving NAC

In this group of 144 breast cancer patients who underwent NAC, we evaluated the association between the TIL score assessed in the pre-NAC biopsy specimen and the patient’s clinical outcome. Kaplan–Meier curves revealed that TILs determined by hot-spot tended to be associated with DFS after surgery for breast cancer patients who underwent NAC (*p* = 0.05, Supplementary Fig. 2a), while there was no significant association between TILs determined by the average method and DFS (*p* = 0.34, Supplementary Fig. 2b). By subtype, there was no association between TILs determined by hot-spot or average methods and DFS (Supplementary Fig. 2c–j). Moreover, although there was no significant association between TILs determined by hot-spot and OS for these patients (Supplementary Fig. 3a), TILs determined by the average method tended to show an association with OS (*p* = 0.08, Supplementary Fig. 3b). Furthermore, by subtype, there was no association between TILs determined by the hot-spot or average methods and OS (Supplementary Fig. 3c–j).

**Fig. 3:**
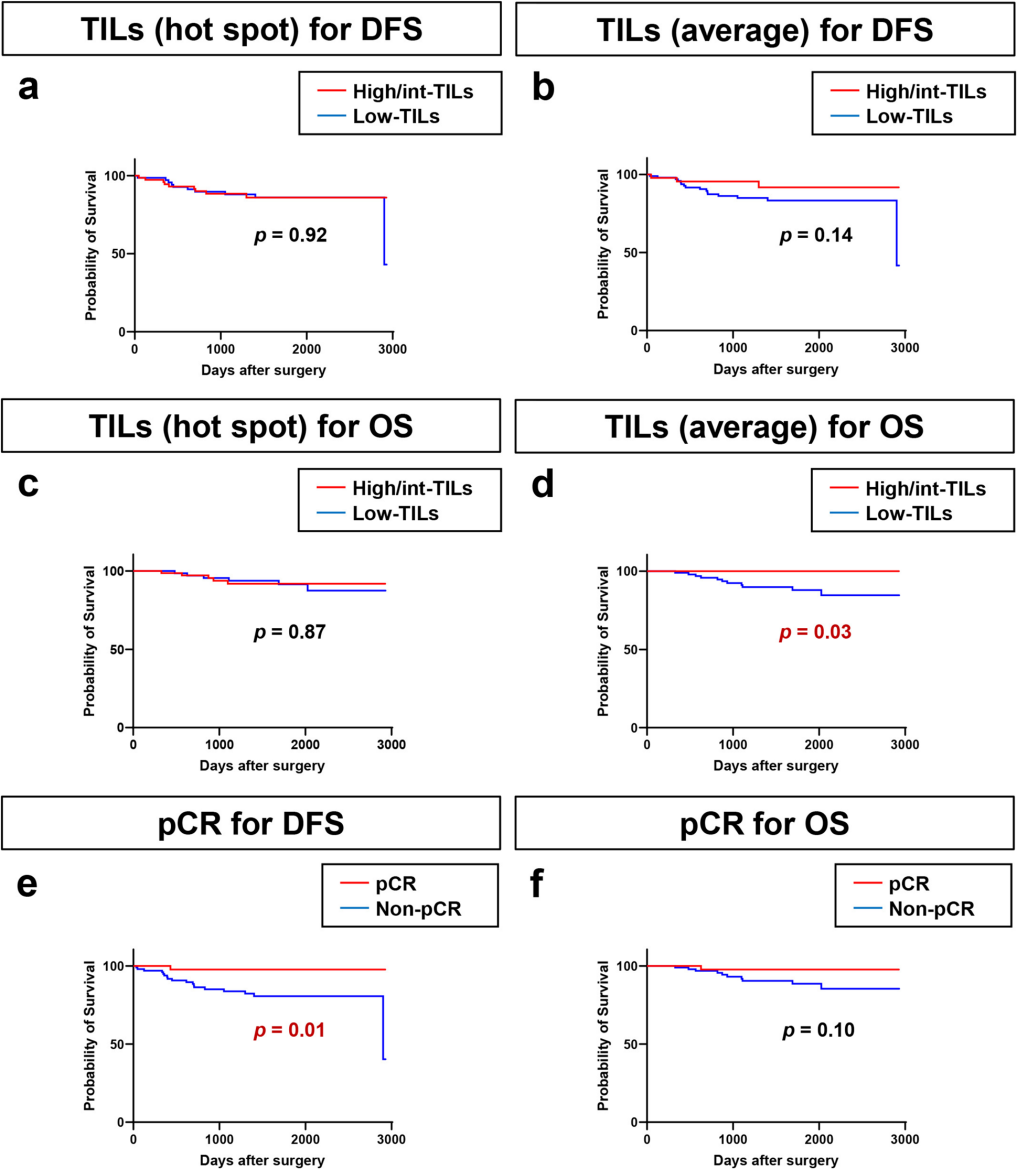
Kaplan–Meier curves showing the impact of pre-treatment tumor-infiltrating lymphocytes (TILs) and pathological complete response (pCR) on the prognosis of 144 patients receiving neoadjuvant chemotherapy. Kaplan–Meier curves showing **a** disease-free survival (DFS) by TIL score (high/intermediate vs. low) using the hot-spot method; **b** DFS by TIL score (high/intermediate vs. low) using the average method; **c** overall survival (OS) by TIL score (high/intermediate vs. low) using the hot-spot method; **d** OS by TIL score (high/intermediate vs. low) using the average method; **e** DFS by pCR status; and (d) OS by pCR status

**Fig. 4:**
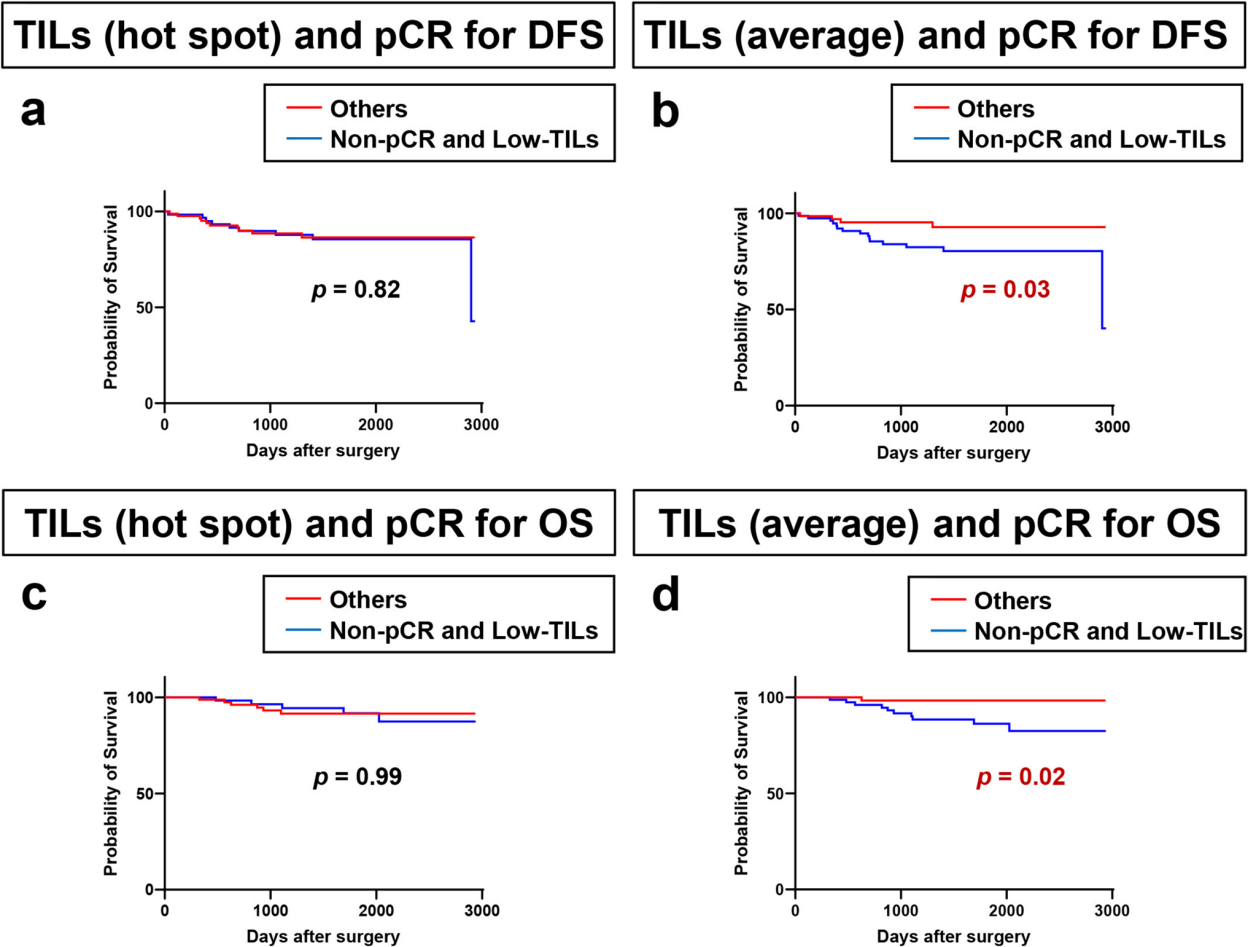
Kaplan–Meier curves showing the impact of the combination of pre-treatment tumor-infiltrating lymphocytes (TILs) and pathological complete response (pCR) on the prognosis of 144 patients receiving neoadjuvant chemotherapy. Kaplan–Meier curves showing **a** disease-free survival (DFS) by TIL score (hot-spot method) and pCR status; **b** DFS by TIL score (average method) and pCR status; **c** overall survival (OS) by TIL score (hot-spot method) and pCR status; and **d** OS by TIL score (average method) and pCR status

Looking carefully at the OS data, all patients with high or intermediate TILs by the average method survived (Supplementary Fig. 3b). Therefore, we re-evaluated the clinical outcomes (DFS and OS) between high/intermediate-TILs and low-TILs for both methods to assess the clinical significance of TILs (Fig. 3a–d). The high/intermediate-TIL group showed a trend toward better DFS (*p* = 0.14, Fig. 3b) and significantly better OS (*p* = 0.03, Fig. 3d) than the low-TIL group by the average method, although analysis using the hot-spot data did not show any significant difference either in DFS or OS (Fig. 3a, c). Next, we confirmed the association between pCR and clinical outcomes; breast cancer patients who achieved pCR had significantly better DFS (*p* = 0.01; Fig. 3e) and tended to have better OS (*p* = 0.10; Fig. 3f) than those who did not achieve pCR. We further hypothesized that combining pCR status with TIL scores might better predict clinical outcomes, and we investigated the relationship between the combined index of TILs/pCR and clinical outcomes (DFS and OS) (Fig. 4). This additional analysis revealed that, by the average method, patients not achieving pCR with low-TILs had significantly poorer DFS and OS than other patients (*p* = 0.03, *p* = 0.02, respectively; Fig. 4b, d), although analysis using the hot-spot data did not show any significant difference in either DFS or OS (Fig. 4a, c). Furthermore, when we analyzed OS by TILs in the patients with recurrence among those receiving NAC, all the patients in the high/intermediate-TILs group were alive, and deaths only occurred in the low-TILs group (Supplementary Fig. 4).

In the 477 eligible surgical cases without NAC, we also examined the difference in clinical outcomes (DFS and OS) between the two groups of high/intermediate and low TILs by the hot-spot and average methods (Supplementary Fig. 5). Although TIL scores by both the hot-spot and average methods did not show any association with DFS, high/intermediate TILs showed significantly better OS than low TILs by the hot spot method (*p* = 0.04, Supplementary Fig. 5c), and high/intermediate TILs tended to have better OS than low TILs by the average method (*p* = 0.11, Supplementary Fig. 5d).

### Prediction of TIL score by machine learning using clinicopathological data without pathological evaluation of TILs

**Fig. 5:**
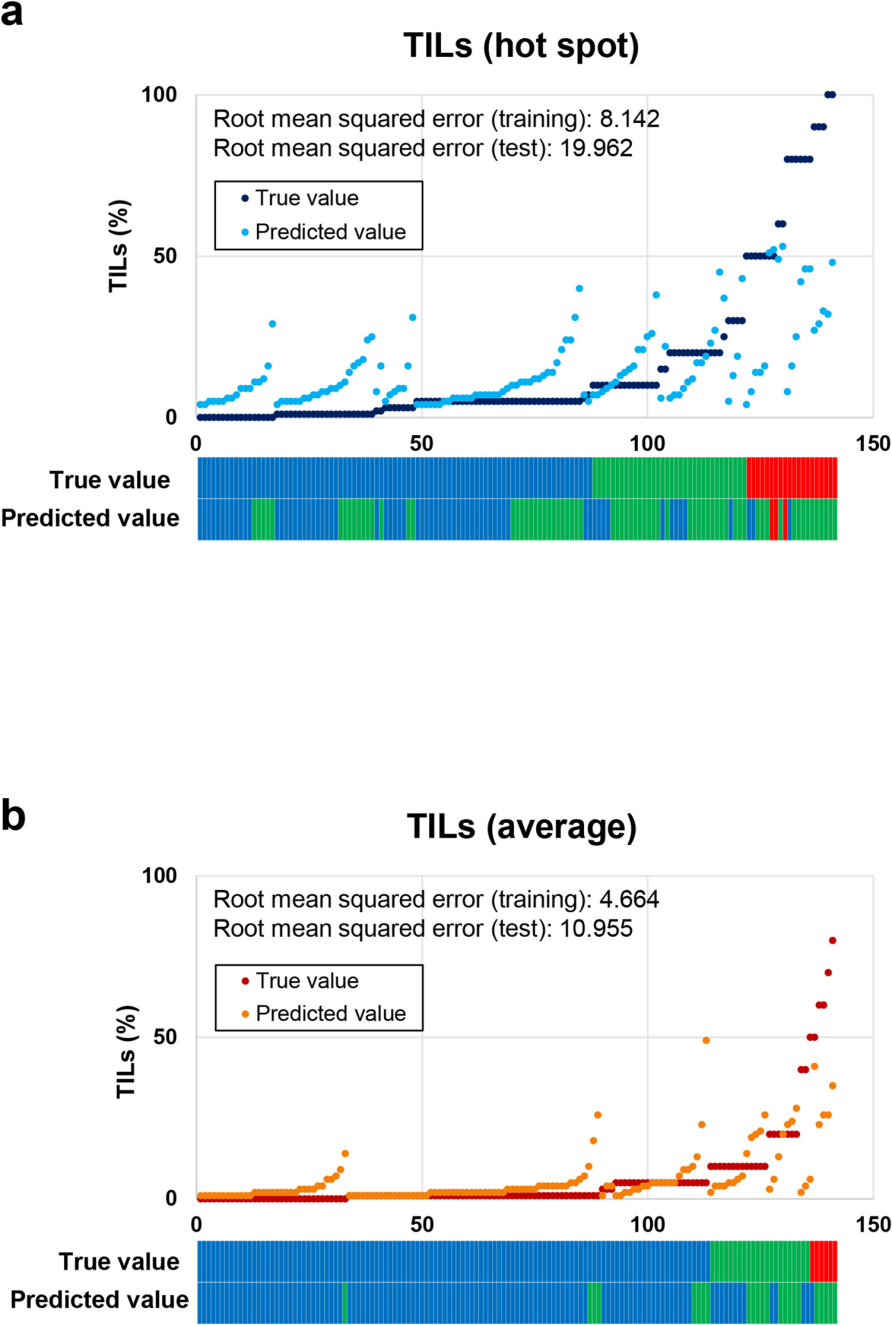
True and predictive values of tumor-infiltrating lymphocytes (TILs) by random forest regression analysis. **a** True values of TIL scores determined by the hot-spot method and predictive values of TIL scores by random forest regression. **b** True values of TIL scores determined by the average method and predictive values of TIL scores by random forest regression. The bar graphs at the bottom of both panels show the TIL categories for the true and predicted values, with red indicating values classified as high-TILs, green as intermediate-TILs, and blue as low-TILs

In patients who received NAC, our data confirmed that the TIL score is related to pCR rate and OS. However, to assess the TIL score for each patient in clinical practice, a significant effort would be required for pathologists, which may limit its implementation. Therefore, we conducted an exploratory study to see if it was possible to predict the TIL score using only clinical parameters obtained in routine clinical practice, without the pathological assessment of TILs. To this end, we used 468 surgical cases to predict TIL scores by machine learning using clinicopathological findings; seventy percent of the cases were used as a training set and thirty percent were used as a test set. TIL scores evaluated by the hot-spot method were predicted using a random forest regressor, and the root mean squared error for the training set was 8.143, and that for the test set was 19.962 (Fig. 5a). Finally, TIL scores evaluated by the average method were predicted using a random forest regressor, and the root mean squared error for the training set was 4.664, and that for the test set was 10.955 (Fig. 5b).

## Discussion

In this study, we demonstrated the usefulness of two different methods of TIL assessment, the hot-spot method and the average method, using biopsy specimens from breast cancer patients treated with NAC. We found that TIL scores obtained by both methods were associated with the pCR rate, suggesting that both methods will be clinically useful. Furthermore, our results suggest that the average method may have an advantage in this setting, as the TIL score by the average method is more consistent between biopsy and surgical specimens, and the TIL score by this method associated better with clinical outcomes compared with the hot-spot method. We also explored the possibility of predicting TIL scores from clinical information in this study and found that machine learning using clinicopathological data may better predict TIL scores assessed by the average method than those assessed by the hot-spot method.

TIL scores obtained by both hot-spot and average methods were linearly related to pCR rates in our 144 breast cancer patients treated with NAC. The relationship between increased TIL scores and high pCR rates has been reported many times in previous studies, especially in HER2-positive subtypes and TNBC [5, 25–29]. Furthermore, in the current study, the combination of pCR and TIL score by the average method related well with clinical outcomes, and patients with low TILs not achieving a pCR were particularly prone to recurrence and death. In breast cancer patients, TILs correlate well, not only with pCR rates, but also with clinical outcomes [30, 31]. In the current study, breast cancer patients who achieved pCR had significantly better DFS and tended to have better OS than those who did not achieve pCR. This suggests that pCR is associated with whether or not the disease recurs. In contrast, TILs are better associated with OS than DFS (Fig. 3b, d), which may suggest that TILs are also associated with outcome after relapse. Indeed, when we looked at the data only for patients who relapsed after NAC, OS appeared to be better when TILs were high/intermediate (Supplementary Fig. 4), suggesting that the treatment efficacy after relapse may be influenced by TILs. Even in the analysis of the surgical cases without NAC, TILs correlated better with OS than with DFS (Supplementary Fig. 5), which may suggest that post-relapse outcomes differ by TILs. TILs have been reported to be associated with treatment outcome not only in patients receiving NAC, but also in patients with metastatic breast cancer [32, 33]. Taken together, it appears that TILs are not only a factor associated with relapse, but also a factor associated with treatment outcome, which we believe is one reason why TILs are better associated with OS than DFS.

Our data suggest that TIL evaluation using the average method may have an advantage for patients treated with NAC, since the TIL score by the average method is more consistent between biopsy and surgical specimens, and the TIL score by this method relates better with clinical outcomes. Due to the nature of the hot-spot method, the larger the area to be evaluated, the greater the number of hot spots and consequently the higher the TIL score. Therefore, it is not surprising that in our results the TIL score by hot-spot was higher in surgical specimens than in biopsy specimens for patients undergoing surgery without preoperative treatment. The current study of patients who received preoperative chemotherapy confirmed the usefulness of the averaging method, given the high proportion of HER2-positive and TNBC patients with relatively high TILs. When studying hormone receptor-positive breast cancer with low overall TILs, the advantages of the hot-spot method should also be kept in mind, as this technique may be able to detect small differences in TILs, and thus assess the patient's condition [17].

TILs can be clinically useful, but it may be practically difficult for pathologists to assess TIL scores in all patients with breast cancer. We explored the possibility of predicting the TIL score from clinical data obtained in routine daily clinical practice, such as blood and biochemical data and pathological findings, and the results suggested that machine learning using clinicopathological data may better predict TIL scores assessed by the average method than those assessed by the hot-spot method. Some researchers are of the opinion that it is difficult to infer TILs and the tumor microenvironment from accessible blood and other systemic data; others indicate it is possible to predict the tumor microenvironment by radiological data [34]. Our results suggest the possibility of indirectly inferring the tumor microenvironment from the analysis of appropriate clinical data from routine pathological diagnosis and laboratory tests, which are widely available. The prediction model we used in this study is lightweight and can be run on ordinary computers, and prediction software based on such a simple model could be developed and widely used in medical facilities. If a more accurate TIL prediction model for clinical use is desired, perhaps the use of imaging data would be ideal, albeit requiring more powerful computers, and further technological advances would be needed to implement such a prediction model widely in society. In the future, it is conceivable that combining these data with imaging and other data may allow TILs to be assessed with a higher degree of accuracy without directly looking at the tumor tissue.

This study had limitations; it was a single-center retrospective study, and the number of patients treated with NAC, in particular, was limited. Regarding machine learning, the predictive ability of the random forest method was demonstrated, but it is clear that further improvement in accuracy is needed before it can be used in clinical practice. However, by using a large number of surgical cases without NAC, the present study clarified the differences between the two TIL scoring methods in the evaluation of biopsy and surgical specimens and showed that both scoring methods are clinically beneficial. Furthermore, our results demonstrated the possibility of inferring the TIL score from clinical information which is of value for the future development of the field.

In conclusion, TIL scores evaluated by two different methods, the average and hot-spot methods, were associated with pCR, suggesting that both are clinically useful as predictive markers for breast cancer patients treated with NAC. The average method may have an advantage for these patients, since the TIL score using the average method is more consistent between biopsy and surgical specimens, and the TIL score by this method relates better with clinical outcomes. Furthermore, our exploratory study shows that machine learning using clinicopathological data may better predict TIL scores assessed by the average method, suggesting the possibility of indirectly inferring the tumor microenvironment from the analysis of appropriate clinical datasets in the future.

## Supplementary Information

Below is the link to the electronic supplementary material.

Supplementary file1 (PDF 84 KB)

Supplementary file2 Supplementary Fig. 1 The percentages of patients with low (blue), intermediate (green) or high (red) tumor-infiltrating lymphocyte (TIL) scores in biopsy specimens using the hot-spot and average methods. Supplementary Fig. 2 Kaplan-Meier curves showing disease-free survival by pre-treatment tumor-infiltrating lymphocyte (TIL) scores by hot-spot and average methods. Kaplan-Meier curves showing disease-free survival by (a) TIL score (hot-spot) for all 144 patients; (c-f) TIL score (hot-spot) for each subtype; (b) TIL score (average) for all 144 patients; and (g-j) TIL score (average) for each subtype. Int intermediate, TNBC triple-negative breast cancer, HER2 human epidermal growth factor receptor 2. Supplementary Fig. 3 Kaplan-Meier curves showing overall survival by pre-treatment tumor-infiltrating lymphocyte (TIL) scores by hot-spot and average methods. Kaplan-Meier curves showing overall survival by (a) TIL score (hot-spot) for all 144 patients; (c-f) TIL score (hot-spot) for each subtype; (b) TIL score (average) for all 144 patients; and (g-j) TIL score (average) for each subtype. Int intermediate, TNBC triple-negative breast cancer, HER2 human epidermal growth factor receptor 2. Supplementary Fig. 4 Kaplan-Meier curves showing the impact of pre-treatment tumor-infiltrating lymphocytes (TILs) on the prognosis of patients with recurrence among those receiving neoadjuvant chemotherapy. Kaplan-Meier curves showing overall survival (OS) by TIL score (high/intermediate vs. low) using the average method. Supplementary Fig. 5 Kaplan-Meier curves showing the impact of tumor-infiltrating lymphocytes (TILs) on the prognosis of 477 surgical cases without neoadjuvant chemotherapy. Kaplan-Meier curves showing (a) disease-free survival (DFS) by TIL score (high/intermediate vs. low) using the hot-spot method; (b) DFS by TIL score (high/intermediate vs. low) using the average method; (c) overall survival (OS) by TIL score (high/intermediate vs. low) using the hot-spot method; and (d) OS by TIL score (high/intermediate vs. low) using the average method (PDF 117 KB)

## Data Availability

The datasets used during the current study are available from the corresponding author on reasonable request.
