# Supplementary material for: Clinical utility of tumor-infiltrating lymphocyte evaluation by two different methods in breast cancer patients treated with neoadjuvant chemotherapy
Source: Breast Cancer. 2025 Jan 14;32(2):404–15. doi: 10.1007/s12282-025-01665-y (PMC11842476; doi:10.1007/s12282-025-01665-y)
Supplement: Supplementary file 1 — Supplementary file1 (PDF 84 KB) [file 12282_2025_1665_MOESM1_ESM.pdf]

Supplementary Table 1

Supplementary Table 1. Conversion of T and N from TNM classification to numerical values

pT value	pN value	Converted into
pT1mi	pN0	0
pT1a	pN1mi	1
pT1b	pN1* (others than pN1mi)	2
pT1c	pN2	3
pTX (X ≧ 2)		X + 2
	pNX (X ≧ 3)	X + 1
