# Supplementary material for: Clinical utility of tumor-infiltrating lymphocyte evaluation by two different methods in breast cancer patients treated with neoadjuvant chemotherapy
Source: Breast Cancer. 2025 Jan 14;32(2):404–15. doi: 10.1007/s12282-025-01665-y (PMC11842476; doi:10.1007/s12282-025-01665-y)

Supplementary Fig. 1

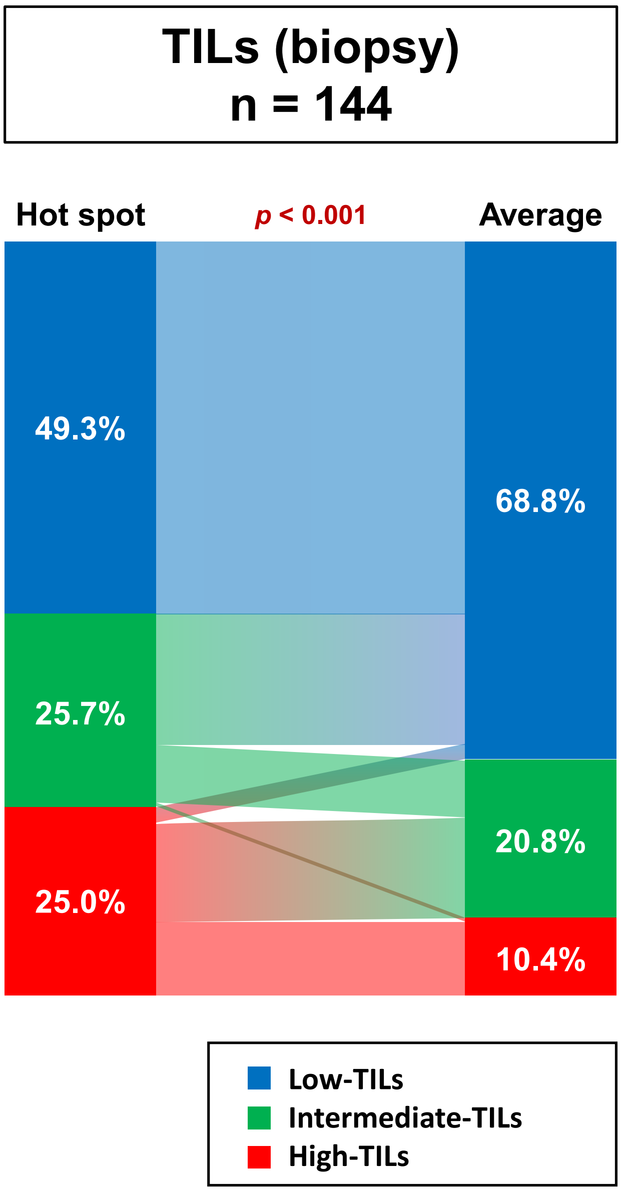

Supplementary Fig. 2

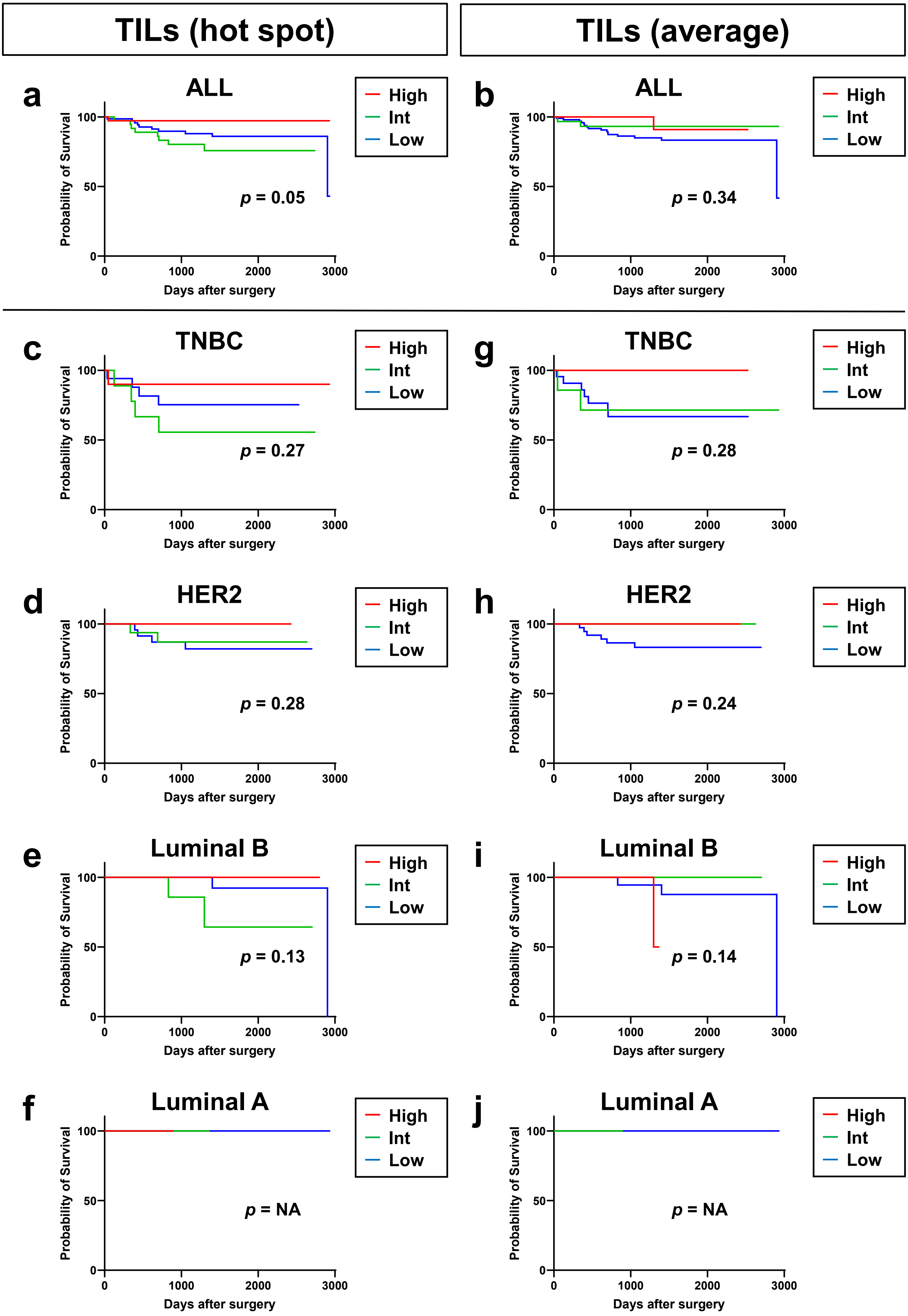

Supplementary Fig. 3

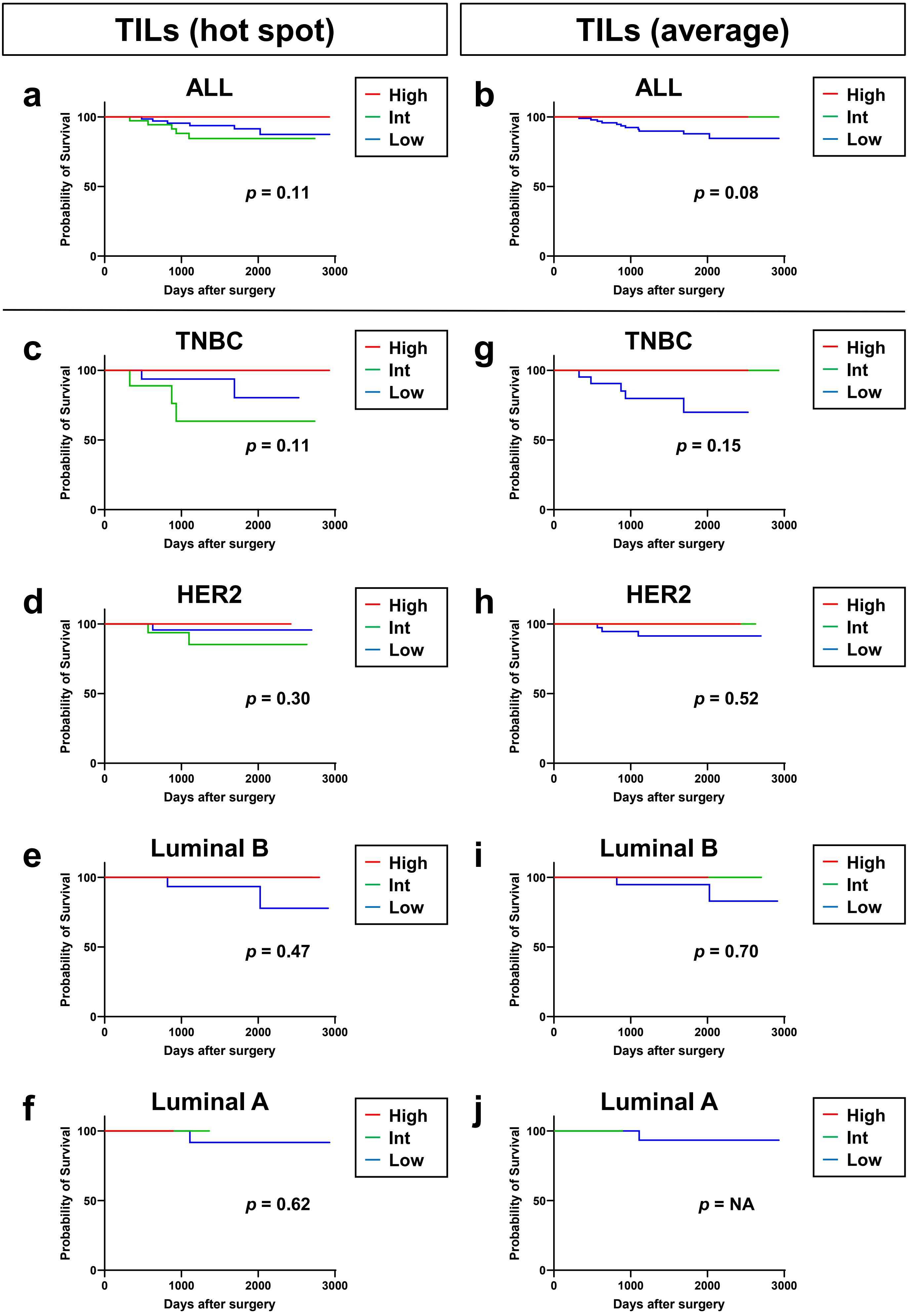

Supplementary Fig. 4

OS in patients with recurrence

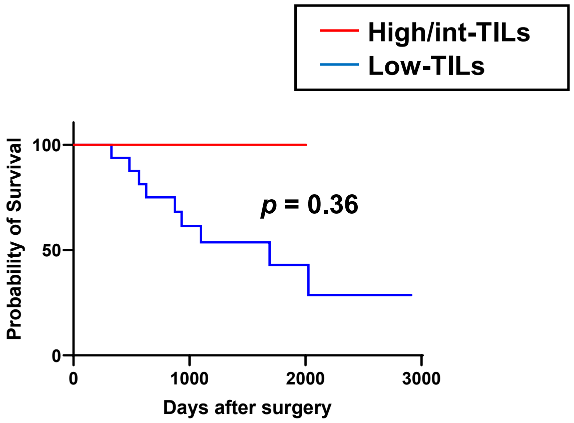

Supplementary Fig. 5

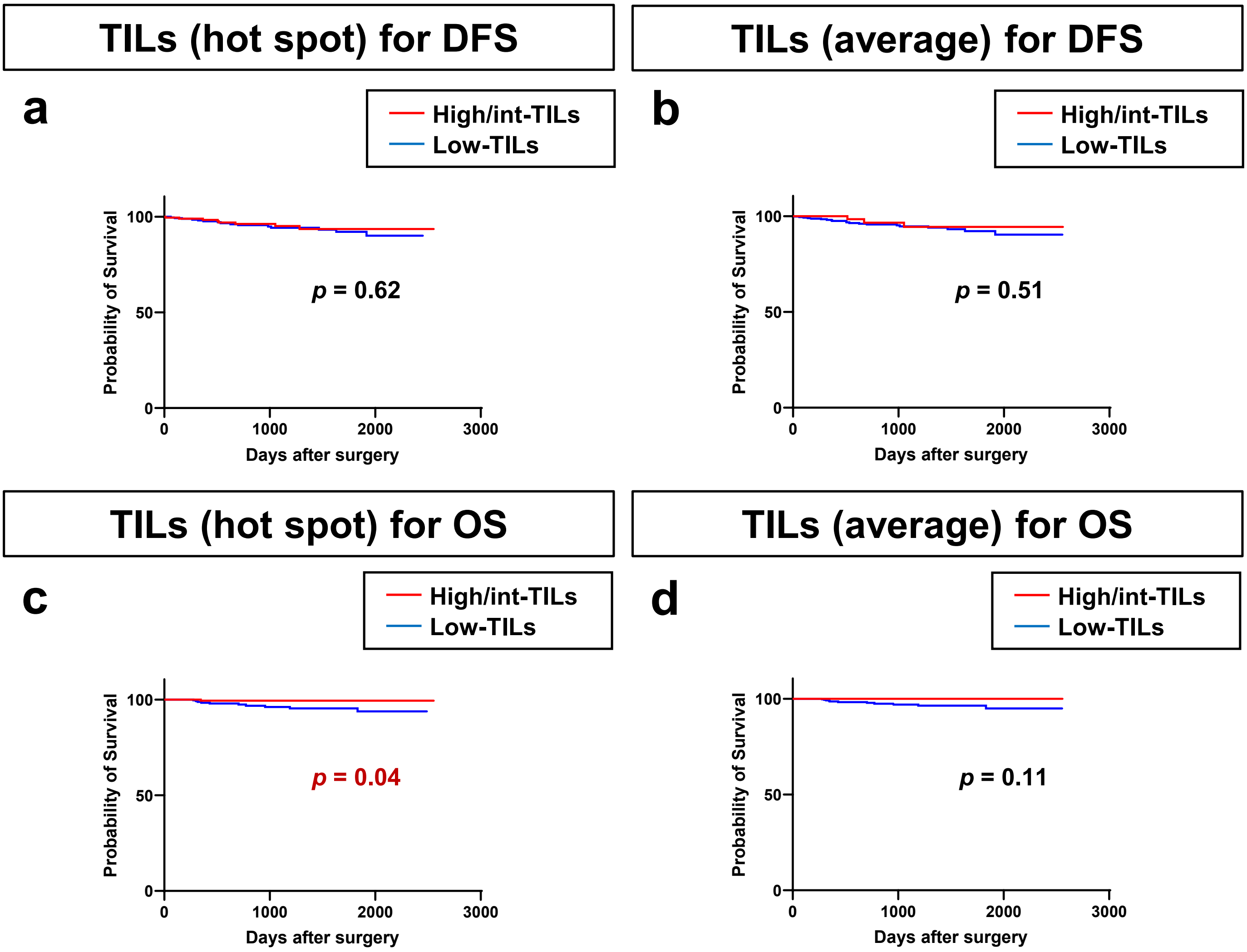

Supplement: Supplementary file 2 — Supplementary file2 Supplementary Fig. 1 The percentages of patients with low (blue), intermediate (green) or high (red) tumor-infiltrating lymphocyte (TIL) scores in biopsy specimens using the hot-spot and average methods. Supplementary Fig. 2 Kaplan-Meier curves showing disease-free survival by pre-treatment tumor-infiltrating lymphocyte (TIL) scores by hot-spot and average methods. Kaplan-Meier curves showing disease-free survival by (a) TIL score (hot-spot) for all 144 patients; (c-f) TIL score (hot-spot) for each subtype; (b) TIL score (average) for all 144 patients; and (g-j) TIL score (average) for each subtype. Int intermediate, TNBC triple-negative breast cancer, HER2 human epidermal growth factor receptor 2. Supplementary Fig. 3 Kaplan-Meier curves showing overall survival by pre-treatment tumor-infiltrating lymphocyte (TIL) scores by hot-spot and average methods. Kaplan-Meier curves showing overall survival by (a) TIL score (hot-spot) for all 144 patients; (c-f) TIL score (hot-spot) for each subtype; (b) TIL score (average) for all 144 patients; and (g-j) TIL score (average) for each subtype. Int intermediate, TNBC triple-negative breast cancer, HER2 human epidermal growth factor receptor 2. Supplementary Fig. 4 Kaplan-Meier curves showing the impact of pre-treatment tumor-infiltrating lymphocytes (TILs) on the prognosis of patients with recurrence among those receiving neoadjuvant chemotherapy. Kaplan-Meier curves showing overall survival (OS) by TIL score (high/intermediate vs. low) using the average method. Supplementary Fig. 5 Kaplan-Meier curves showing the impact of tumor-infiltrating lymphocytes (TILs) on the prognosis of 477 surgical cases without neoadjuvant chemotherapy. Kaplan-Meier curves showing (a) disease-free survival (DFS) by TIL score (high/intermediate vs. low) using the hot-spot method; (b) DFS by TIL score (high/intermediate vs. low) using the average method; (c) overall survival (OS) by TIL score (high/intermediate vs. low) using the [file 12282_2025_1665_MOESM2_ESM.pdf]
